# Investigating the causal pathways among psychopathological variables, cognitive impairment, and real-life functioning in people with schizophrenia

**DOI:** 10.1192/j.eurpsy.2025.890

**Published:** 2025-08-26

**Authors:** E. Caporusso, L. Giuliani, F. Sanmarchi, A. Mucci, P. Rucci, P. Bucci, G. M. Giordano, M. Amore, P. Rocca, A. Rossi, A. Bertolino, S. Galderisi, M. Maj

**Affiliations:** 1 Department of Mental and Physical Health and Preventive Medicine, University of Campania “Luigi Vanvitelli”, Naples; 2 Department of Biomedical and Neuromotor Sciences, University of Bologna, Bologna; 3 Department of Neurosciences, Rehabilitation, Ophthalmology, Genetics and Maternal and Child Health, Section of Psychiatry, University of Genoa, Genoa; 4 Department of Neuroscience, Section of Psychiatry, University of Turin, Turin; 5 Department of Biotechnological and Applied Clinical Sciences, Section of Psychiatry, University of L’Aquila, L’Aquila; 6 Department of Basic Medical Science, Neuroscience and Sense Organs, University of Bari ‘Aldo Moro’, Bari, Italy

## Abstract

**Introduction:**

The present study aimed to investigate the causal relationships among cognitive impairment, psychopathology, and real-life functioning in a large sample of people with schizophrenia, using a data-driven causal discovery procedure based on partial ancestral graphs (PAGs).

**Objectives:**

This method may provide additional insights for identifying potential targets of therapeutic interventions to promote recovery in people with chronic schizophrenia.

**Methods:**

State-of-the-art instruments were used to assess the study variables. Two PAGs were generated at baseline and after 4 years of follow-up to explain the nature of the causal relationships linking psychopathology, cognition, and functioning.

**Results:**

The study sample was composed of 612 clinically stable patients with schizophrenia at baseline and 602 at follow-up. The PAGs suggested that working memory deficit is the first ancestor of the causal links, influencing all the other neurocognitive domains, social cognition, and functional capacity, which in turn affects everyday life functioning. From this domain of functioning a causal link is directed to disorganization and positive symptoms, and another to work skills and interpersonal relationships domains; the latter had a direct link to asociality and the other domains of negative symptoms. The structure of the PAGs did not differ significantly between baseline and follow-up, indicating the stability of the causal relationships.

**Conclusions:**

The role of working memory deficits in the pathways to functional outcomes in schizophrenia highlights the importance of implementing integrated pharmacological and cognitive remediation interventions targeting neurocognition. The impact of everyday life and interpersonal functioning on the clinical presentation of schizophrenia suggests that integrated and personalized treatments, promoting relevant skills to improve these functional outcomes, may have a beneficial impact on clinical outcomes.

**Disclosure of Interest:**

None Declared

